# An SNN retrocopy insertion upstream of *GPR22* is associated with dark red coat color in Poodles

**DOI:** 10.1093/g3journal/jkac227

**Published:** 2022-09-01

**Authors:** Kevin Batcher, Scarlett Varney, Verena K Affolter, Steven G Friedenberg, Danika Bannasch

**Affiliations:** Department of Population Health and Reproduction, University of California, Davis, Davis, CA 95616, USA; Department of Population Health and Reproduction, University of California, Davis, Davis, CA 95616, USA; Department of Pathology, Microbiology, & Immunology, University of California, Davis, Davis, CA 95616, USA; Department of Veterinary Clinical Sciences, University of Minnesota, St Paul, MN 55455, USA; Department of Population Health and Reproduction, University of California, Davis, Davis, CA 95616, USA

**Keywords:** pheomelanin, coat color, canine, retrogene, GWAS, inherited, dog

## Abstract

Pigment production and distribution is controlled through multiple genes, resulting in a wide range of coat color phenotypes in dogs. Dogs that produce only the pheomelanin pigment vary in intensity from white to deep red. The Poodle breed has a wide range of officially recognized coat colors, including the pheomelanin-based white, cream, apricot, and red coat colors, which are not fully explained by the previously identified genetic variants involved in pigment intensity. Here, a genome-wide association study for pheomelanin intensity was performed in Poodles which identified an association on canine chromosome 18. Whole-genome sequencing data revealed an *SNN* retrocopy insertion (*SNNL1*) in apricot and red Poodles within the associated region on chromosome 18. While equal numbers of melanocytes were observed in all Poodle skin hair bulbs, higher melanin content was observed in the darker Poodles. Several genes involved in melanogenesis were also identified as highly overexpressed in red Poodle skin. The most differentially expressed gene however was *GPR22*, which was highly expressed in red Poodle skin while unexpressed in white Poodle skin (log_2_ fold change in expression 6.1, *P* < 0.001). *GPR22* is an orphan G-protein-coupled receptor normally expressed exclusively in the brain and heart. The *SNNL1* retrocopy inserted 2.8 kb upstream of *GPR22* and is likely disrupting regulation of the gene, resulting in atypical expression in the skin. Thus, we identify the *SNNL1* insertion as a candidate variant for the CFA18 pheomelanin intensity locus in red Poodles.

## Introduction

In dogs, as with other mammals, coat color patterns are the result of varied production of the yellow-red pigment, pheomelanin, and the black pigment, eumelanin. While most dogs produce a mixture of both pigments, loss of function mutations in the pigment-type switching genes melanocortin 1 receptor (*MC1R*) and agouti signaling protein result in production of only 1 pigment type (Newton *et al.* 2000; Kerns *et al.* 2004; Berryere *et al.* 2005). Among pheomelanin-based dogs, pigment intensity can vary greatly within and between breeds, from white to deep red (Sponenberg and Rothschild 2001). Multiple genetic variants that modify pheomelanin pigment intensity have been identified in dogs, highlighting the complex, multigenic nature of coat color phenotypes. A missense variant in the *MFSD12* gene and a copy number variant near *KITLG* have both been associated with pheomelanin intensity in a variety of breeds (Hédan *et al.* 2019; Weich *et al.* 2020). An across breed analysis of pheomelanin intensity that was published while the current study was being performed identified that genetic variants at 5 loci explained 70% of pheomelanin intensity in dogs, which included the variants at *MFSD12* and *KITLG* as well as 3 novel loci on canine chromosomes (CFA) 2, CFA18, and CFA21 (Slavney *et al.* 2021). However, it is unclear how much each of the loci contribute to pheomelanin intensity within individual breeds.

The Poodle breed has 3 size varieties (toy, miniature, and standard) and 11 coat colors that are officially recognized by the American Kennel Club, 4 of which are pheomelanin-based: white, cream, apricot, and red (www.akc.org). While the *MFSD12* dilution variant was present in the white Poodles, it alone does not explain the range of pheomelanin intensity between the cream, apricot, and red Poodles (Hédan *et al.* 2019). Additionally, the copy number variant near *KITLG*, which was associated with pigment intensity in the pheomelanin-based Nova Scotia Duck Tolling Retrievers and the eumelanin-based silver and black Poodles, was not found to be associated with pigment intensity between the pheomelanin-based white and red Poodles, indicating that additional genetic factors affecting pheomelanin intensity exist within the Poodle breed (Weich *et al.* 2020).

In this study, the genetics of pheomelanin intensity was analyzed within a single breed, the Poodle. A quantitative genome-wide association study (GWAS) was performed and a single associated locus on CFA18 was identified. An *SNN* gene retrocopy insertion was then identified as the most likely causative variant behind pheomelanin intensity in Poodles.

## Methods

### Sample collection

Collection of all Poodle samples (*N* = 225) was approved by the University of California, Davis Animal Care and Use Committee (protocol #18561). Breed, date of birth, sex, weight, and color were reported by the owner. Owners provided whole blood or buccal swabs from their privately owned dogs (58 white, 17 cream, 3 apricot, 6 red) in collaboration with the Poodle Club of America Foundation (grant #A182159001), and DNA was extracted using a Gentra Puregene DNA extraction kit (Qiagen, Valencia, CA, USA). Additional Poodle DNA samples from the Bannasch lab DNA repository at UC Davis were also included in the study (67 white, 9 cream, 23 apricot, 42 red). RNA from 8 red and 9 white Poodles was extracted from neonatal canine dewclaw samples using an RNeasy Fibrous Tissue Mini Kit (Qiagen, Valencia, CA, USA).

### Genome-wide association

In order to perform a quantitative GWAS, Poodles were designated from 1 to 4 based on owner described coat color (also the AKC registered coat color), with white as “1,” cream as “2,” apricot as “3” and red as “4.” Genome-wide SNV genotyping was performed on the Illumina Canine HD BeadChip array. All dogs were confirmed homozygous for the recessive yellow “e” allele at *MC1R* with the exception of 2 cream Poodles which were heterozygous and thus excluded from further analysis (Newton *et al.* 2000). Variants with a minor allele frequency of less than 5% or less than 90% total genotyping rate were excluded using PLINK, resulting in 163,753 total variants (Purcell *et al.* 2007). The Bonferroni-corrected genome-wide significance threshold was set at *P* = 3.05 × 10^−7^. A multidimensional scaling plot showed that standard Poodles clustered separately from the toy and miniature Poodles, highlighting population stratification in the dataset (Supplementary Fig. 1). To control for this, the GWAS was performed using a univariate mixed model with a standardized relatedness matrix in GEMMA v.0.97 (Zhou and Stephens 2012). A similar GWAS using only the miniature and toy Poodles (*N* = 57) was also performed using GEMMA.

### Variant detection

Whole-genome sequencing (WGS) data from standard Poodles (7 white, 1 apricot, and 1 red) were aligned to UU_Cfam_GSD_1.0 (Wang *et al.* 2021) using BWA v0.717 and converted to BAM files using samtools v1.14, both with default parameter settings (Li 2009). Variant calling across the critical interval was performed using bcftools mpileup (Danecek *et al.* 2021). Based on the GWAS results, the assumed inheritance pattern was alternate homozygotes for the red and white Poodles and heterozygous for the apricot Poodle. The WGS samples were confirmed to match this pattern at the top 4 GWAS SNV. Variants were tested for function using the Ensembl variant effect predictor (McLaren *et al.* 2016) with the UU_Cfam_GSD_1.0 annotation. Missense variants were tested for function using SIFT and Polyphen-2 (Ng and Henikoff 2003; Adzhubei *et al.* 2010). The region was also analyzed for structural variants through visual analysis of the alignment files of a single red Poodle in comparison to a single white Poodle using Integrative Genomics Viewer (IGV) (Robinson *et al.* 2011). To maintain consistency with the variants reported from the GWAS, all genomic locations were reported as their location in the CanFam3.1 reference.

### Genotyping and Sanger sequencing

A 3 primer PCR assay was developed for genotyping *SNNL1*, with a forward and reverse primer flanking the insertion site and 1 primer internal to the retrocopy. Internal primers were then used for sequencing the entire retrocopy. Primers were also designed for genotyping the *SLC26A4* chr18:12,910,382 C/T variant. All primers were developed using Primer3 software (Untergasser *et al.* 2012). The primers used in this study are available in Supplementary Table 1. Sanger sequencing was performed on an Applied Biosystems 3500 Genetic Analyzer using a Big Dye Terminator Sequencing Kit (Life Technologies, Burlington, ON, Canada). Additional genotyping of *SNNL1* from WGS data was performed visually in IGV. Samples which had no reads crossing either of the breakends at the insertion site were considered homozygous for the retrocopy insertion.

### Histopathological and immunohistochemical examinations

Submitted skin samples from the dewclaws of a white Poodle (*SNNL1* 0 copies), a cream Poodle (*SNNL1* 1 copy), and a red Poodle (*SNNL1* 2 copies) were used for histopathological analysis. The samples were fixed in 4% buffered formalin, bisected and embedded in paraffin. Five-micron paraffin sections were used for both histopathology and immunohistochemistry. Presence of melanin granules within matrical cells of the hair follicles as well as within hair shafts was assessed by Fontana-Masson’s stain. Anti-Sox10 antibody (mouse monoclonal, Abcam Ref. ab212843), which recognizes cells of neural crest origin, was used to identify melanocytes among the matrical cells within hair bulbs of anagen hair follicles, which are actively forming a new hair shaft. For immunohistochemistry, sections were deparaffinized (xylene: 10 min 2×, followed by 100% ethanol: 1 min 3×, 95% ethanol: 1 min and 70% ethanol: 1 min), followed by quenching of endogenous peroxidase (500 µl 10% sodium azide; 500 µl 30% hydrogen peroxide in 50 ml PBS; 25 min at room temperature) and 3 rinses in PBS. Antigen retrieval was performed by immersing slides in preheated antigen retrieval solution (1× Dako Target Retrieval Solution; stock solution S1699, pH6 at 95–100°C; 5 min). Slides were then cooled down to room temperature and washed 3 times in PBS. After exposing slides to 10% horse serum in PBS (15 min), the anti-Sox10 antibody (mouse monoclonal, Abcam Ref. ab212843) was applied at a 1:100 dilution for 1 h. After 3 rinses in PBS, the following steps were performed: (1) application of ImmPRESS HRP Horse Anti-Mouse IgG Polymer Reagent (Vector Cat. #MP-7402; 30 min), (2) thorough PBS rinses, and (3) addition of substrate (Vector, SK-4800). Development was monitored microscopically and reaction was stopped by immersing the slides in Milli-Q/distilled water. Counterstain in Gill’s hematoxylin #2 (RICCA, 3536-16; 15–30 s) was stopped by washing slides in running tap water. Slides were then cover slipped using Shandon-Mount media (Thermo Scientific, 1900331).

### RNAseq analysis

Poly(A) capture RNAseq Library preparation and NovaSeq S4 Illumina paired end sequencing were performed in 3 red and 1 white Poodle at the UC Davis Genome Center. RNAseq data were aligned to UU_Cfam_GSD_1.0 (Wang *et al.* 2021) using minimap v2.21 (Li 2018). Alignment files were analyzed for evidence of chimeric transcripts using IGV. Batch 3′ TagSeq library preparation and HiSeq 4000 Illumina single-end sequencing were performed on 8 red and 9 white Poodles at the UC Davis Genome Center. TaqSeq generates a single initial library molecule per transcript which is ideal for differential gene expression analysis (Meyer *et al.* 2011). Unique molecular identifiers were removed from the TagSeq data using UMI-tools (Smith *et al.* 2017), and reads were also trimmed to remove Illumina adaptors and polyA read through using bbduk (Bushnell 2014). Reads were aligned to UU_Cfam_GSD_1.0 (Wang *et al.* 2021) using STAR v2.7.9a (Dobin *et al.* 2013). The UU_GSD_1.0 annotation was used to perform gene counting with htseq-count (Anders *et al.* 2015). Genes with overlapping 3′ UTR in the annotation resulted in reads not being counted due to ambiguity; therefore, the “–nonunique all” option was used, which counts ambiguous reads to all overlapping features. Differential gene expression was performed using Limma-Voom (Law *et al.* 2014) and is reported as log_2_ fold change (FC) increase in expression in the red Poodles, where a negative FC indicates higher expression in white Poodles. Genes which had fewer than 5 normalized read counts across all samples were filtered. Different individuals were used in the RNAseq and TagSeq analyses than those used for variant discovery in the WGS analysis.

## Results

### Genome-wide association for pheomelanin intensity in Poodles

**Fig. 1.**
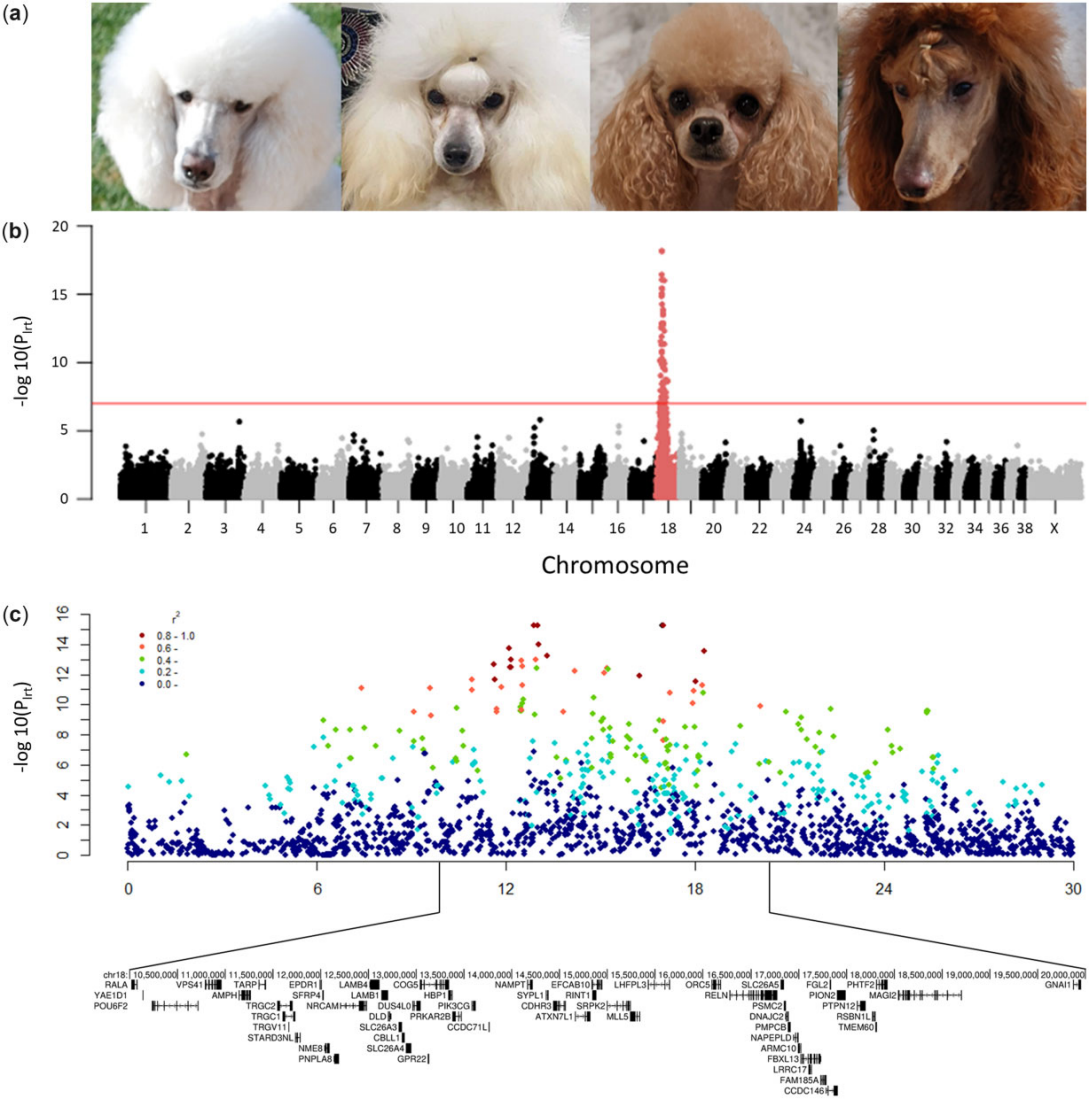
Genome-wide association for red coat color in Poodles. a) Owner provided photographs of a white, cream, apricot, and red Poodles, b) Manhattan plot showing *P* values for likelihood-ratio tests calculated in GEMMA (λ = 1.014). Bonferroni-corrected genome-wide significance is indicated by the solid line. c) The genome-wide significant region on chromosome 18 with nearby SNV color based on LD (*R*^2^) with one of the top associated SNV (chr18:16,968,786).

To identify regions of the genome associated with pheomelanin intensity specific to Poodles, a quantitative GWAS was performed in white (*N* = 51), cream (*N* = 5), apricot (*N* = 15), and red (*N* = 8) Poodles (Fig. 1a). A single locus on chromosome 18 reached genome-wide significance (Fig. 1b). A Q-Q plot of expected and observed chi-squared values indicated that population stratification was successfully controlled for (λ = 1.014; Supplementary Fig. 2). The top 4 associated variants, shown in Table 1, were in near perfect linkage disequilibrium (LD). Analysis of LD between the top associated SNV (chr18:16,968,786) and nearby variants revealed a large region of LD in Poodles (Fig. 1c). To determine if population structure within the dataset was affecting the association, a separate GWAS using only the miniature and toy Poodles (*N* = 57) was performed, which identified the same top 4 SNVs and confirmed the CFA18 association with pheomelanin intensity (Supplementary Fig. 3).

**Table 1.** Top genetic markers associated with red coat color in Poodles (CanFam3.1).

CanFam3.1 position	Red allele	AF white	AF cream	AF apricot	AF red	P_LRT_
chr18:16968786	G	0	0.200	0.500	0.722	5.11 × 10^−23^
chr18:17006104	A	0	0.200	0.500	0.722	5.11 × 10^−23^
chr18:12910382	T	0	0.100	0.500	0.722	6.59 × 10^−23^
chr18:13022106	G	0	0.100	0.500	0.722	6.59 × 10^−23^

AF, allele frequency of the red associated allele in each category.

### Whole-genome sequencing analysis

**Fig. 2.**
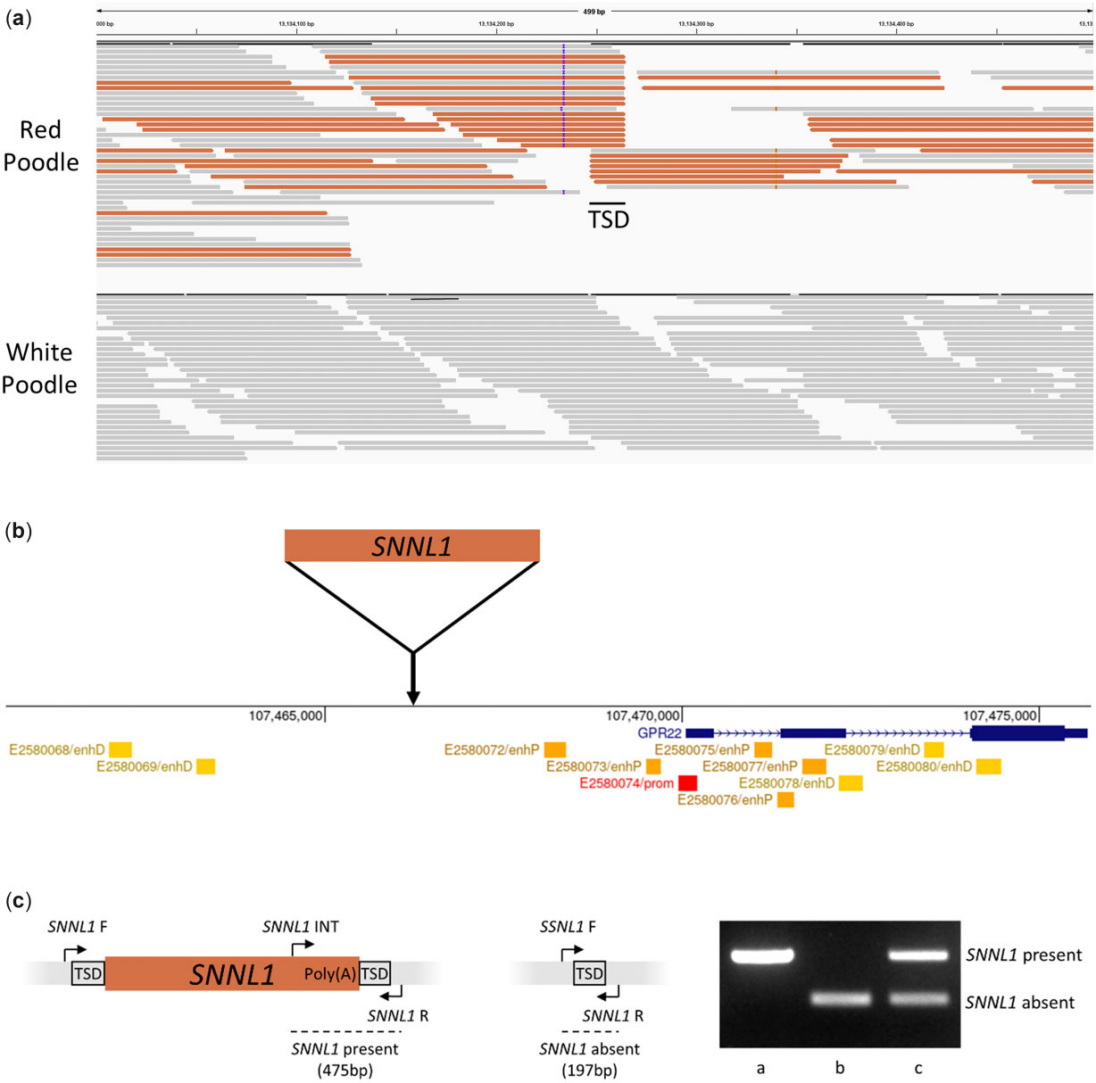
An SNN retrocopy identified in red Poodles. a) A discordant read cluster observed in IGV indicating the presence of an *SNN* retrocopy insertion in a red Poodle (top track) which was absent from white Poodles (bottom track). b) The location of the *SNNL1* retrocopy insertion within the syntenic region in humans. c) Two external primers flanking the *SNNL1* insertion and 1 internal primer are used to genotype *SNNL1.* When *SNNL1* is present, a 475-bp product is observed (a), while a 197-bp product is observed when *SNNL1* is absent (b). Heterozygous individuals have both bands (c).

Variants within the interval of CanFam3.1 chr18:10–20 mb were analyzed from WGS data in 9 standard Poodles (7 white, 1 apricot, 1 red). Out of 47,095 total variants identified, 5,603 segregated by phenotype, including 4,834 SNV and 762 short indels. Variant effect prediction identified missense variants in 5 genes (Table 2), including a previously reported variant in the *SLC26A4* gene (chr18:12,910,382 T > C) that was associated with pheomelanin intensity across breeds (Slavney *et al.* 2021). While the 2 missense variants in *ARMC10* and *GSAP* were predicted to be deleterious by both SIFT and Polyphen-2, none of the missense variants affected genes known to be involved in any pigment pathways. Therefore, visual analysis of the aligned sequence data was also performed to identify larger structural variants. A cluster of discordant reads was observed in the red and apricot Poodles at approximately chr18:13,134,000–13,134,500 which mapped to the *Stannin* (*SNN*) gene locus (chr6:31,137,750–31,147,848), highlighting a putative retrocopy insertion (Fig. 2a).

**Table 2.** Missense variants identified in red Poodles.

CanFam3.1 location	Gene	Amino acid	Codons	dbSNP	SIFT	Polyphen-2
chr18:12529314	*LAMB4*	G/E	gGa/gAa	rs852976135	Tolerated (0.44)	Benign (0.035)
chr18:12910382	*SLC26A4*	I/M	atA/atG	rs852750854	Deleterious (0.03)	Benign (0.014)
chr18:14468631	*CDHR3*	G/R	Ggg/Agg	rs22643100	Tolerated (0.42)	Benign (0.011)
chr18:17006104	*ARMC10*	F/C	tTt/tGt	rs853061060	Deleterious (0)	Damaging (1.0)
chr18:17469333	*GSAP*	D/N	Gat/Aat	rs850968557	Deleterious (0)	Damaging (1.0)

### *SNN* retrocopy analysis

The putative *SNN* retrocopy was investigated using primers flanking the insertion site to PCR amplify the region in a red Poodle. Sanger sequencing confirmed the insertion as a full-length *SNN* retrocopy (Supplementary File 1), referred to here as *SNNL1*. *SNNL1* is inserted within the intron of *COG5* and 2.8 kb upstream of and in the same orientation as *GPR22*. The *SNNL1* retrocopy sequence contains 2 SNV in the 3′ UTR (chr6:31,139,403 C > A and chr6:31,140,045 G > A), but is otherwise identical to the parent gene sequence. *SNNL1* has a 3′ poly (A) tail approximately 27 bp in length, and a 17-bp target site duplication (TGTGAAATACTGAAGTT) was also observed flanking the insertion, putting the exact insertion location at chr18:13,134,248–13,134,264. The syntenic region in humans for *SNNL1* was viewed to determine its location relative to regulatory elements*. SNNL1* inserted 2.8 kb upstream of *GPR22*, nearby multiple predicted *GPR22* enhancers (Fig. 2b).

### Genotyping *SNNL1* and the *SLC26A4* missense variant

A 3 primer PCR genotyping assay was developed for *SNNL1* (Fig. 2c). The retrocopy was then genotyped in a larger dataset of white, cream, apricot, and red Poodles to test the association with coat color (*N* = 224). *SNNL1* copy number was highly predictive of red coat color in the breed (adjusted *R*^2^ = 0.840, *P* = 2.17 × 10^−90^) (Table 3). All (*N* = 125) white Poodles had 0 copies of *SNNL1*, and all red Poodles (*N* = 48) had at least 1 copy of *SNNL1*, with 38/48 of them having 2 copies. Most (19/25) apricot Poodles had 1 copy of *SNNL1*. The allele frequencies were 0.096 in cream, 0.500 in apricot, and 0.896 in red Poodles, indicating an additive effect on pheomelanin intensity. The nearby missense variant in *SLC26A4* (chr18:12,910,382 T > C) was also genotyped in the same set of dogs to access LD in the region, and the “C” allele and the *SNNL1* insertion were found to be in complete LD in the white, apricot, and red Poodles, however, 1 cream Poodle was identified with 0 copies of *SNNL1* that was heterozygous for the *SLC26A4* variant.

**Table 3.** *SNNL1* copy number in white, cream, apricot, and red Poodles.

Coat color	*SNNL1* copy number			Total	Allele frequency
	0	1	2		
White	125	0	0	125	0.000
Cream	21	5	0	26	0.096
Apricot	3	19	3	25	0.500
Red	0	10	38	48	0.896

The linkage between the chr18:12,910,382 T > C variant and *SNNL1* was further assessed in a publicly available WGS dataset (Plassais *et al.* 2019). The “C” allele was observed in Tibetan Mastiffs, Chow Chows, village dogs, and a Xoloitzcuintli, Qingchuan, and Chongqing dog (Supplementary Table 2). *SNNL1* was also genotyped in these same breeds through visual analysis of the aligned WGS data, and while *SNNL1* was in strong LD with chr18:12,910,382 T > C, 8 village dogs and 1 Tibetan Mastiff were identified that have the SNV but do not appear to have *SNNL1*, indicating that linkage between the 2 is incomplete (Supplementary Table 2). Although Tibetan Mastiffs, Chow Chows, Xoloitzcuintli, Qingchuan, and Chongqing dogs all have deep red pheomelanin segregating within the breeds, we did not have access to phenotype data for the dogs from the WGS to confirm any associations.

### Histopathological and immunohistochemical examinations

**Fig. 3.**
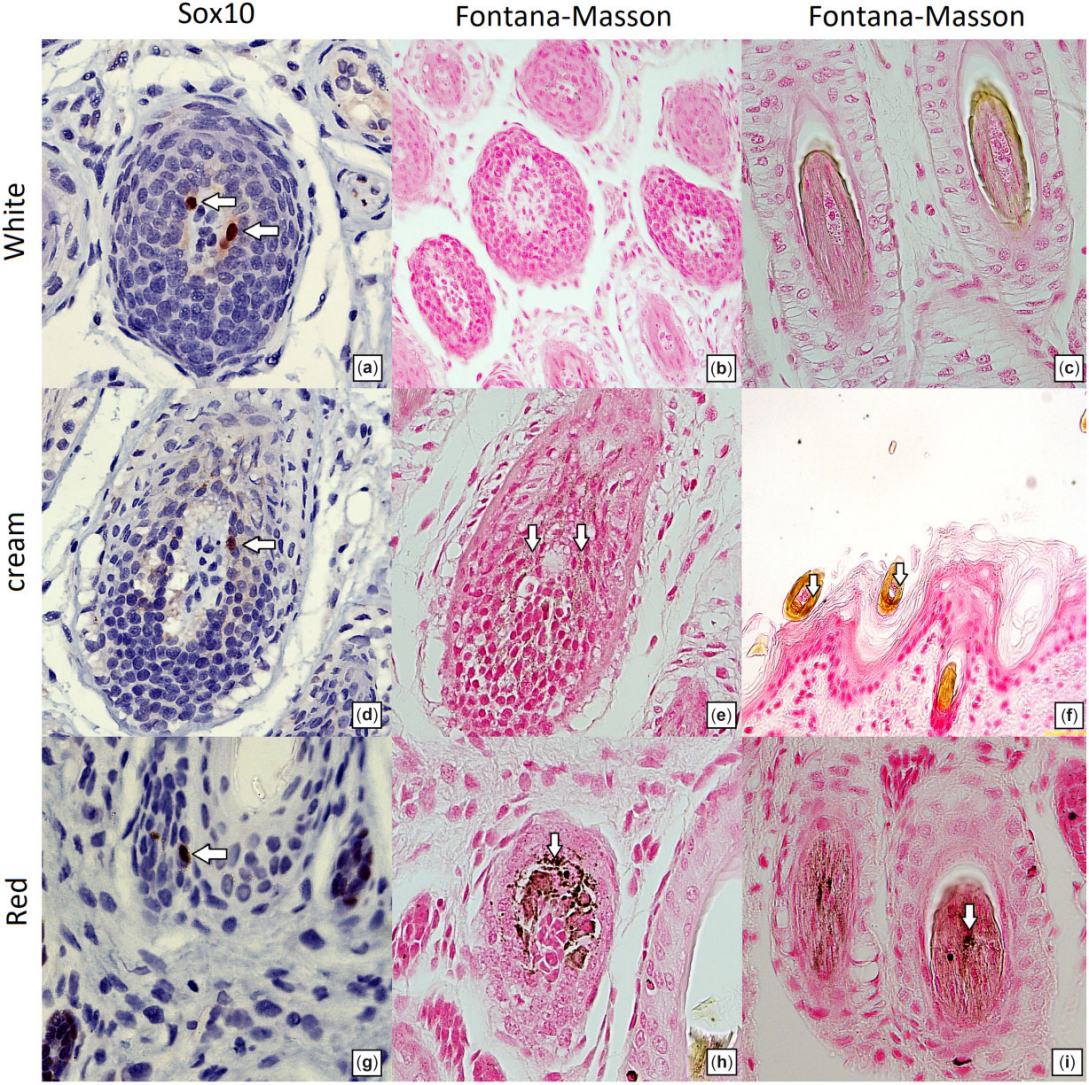
Presence of melanocytes within hair bulbs and melanin granules within matrical cells and hair shafts. Melanocytes were identified by the presence of SOX10, and melanin was identified by Fontana-Masson staining. White Poodle with zero copies of *SNNL1:* despite the presence of melanocytes (a) in the hair bulb, no pigment is noted in the matrical cells of the hair bulb (b) or the cuticle of hair shafts (c). Cream Poodle with 1 copy of *SNNL1*: in addition to melanocytes (d) in the hair bulb, there is fine melanin dusting of matrical cells of the hair bulb (e). The hair shaft cuticle contains melanin granules (f). Red Poodle with 2 copies of *SNNL1:* in addition to melanocytes (g) in the hair bulb, there is marked presence of melanin granules in matrical cells of the hair bulb with melanin pigment (h) as well as in hair shaft cuticle (i).

Histopathological analysis was performed in skin tissue from a white (0 copies *SNNL1*), cream (1 copy *SNNL1*), and red (2 copies *SNNL1*) Poodles. Expression of Sox10, identifying melanocytes within the hair bulb, was observed in all Poodles irrespective of coat color or *SNNL1* copy number (Fig. 3, a, d, and g). However, the white Poodle with 0 copies of *SNNL1* lacked melanin within the hair bulbs and hair shaft cuticle (Fig. 3, b and c). Some melanin was observed in a cream Poodle with 1 copy of *SNNL1* (Fig. 3, e and f), but melanin was most prominent in the red Poodles with 2 copies of *SNNL1* (Fig. 3, h and i). The equivalent melanocytes and differential melanin indicated that the red coat color was occurring due to an increase in pigment synthesis.

### Gene expression analysis in Poodle skin

**Fig. 4.**
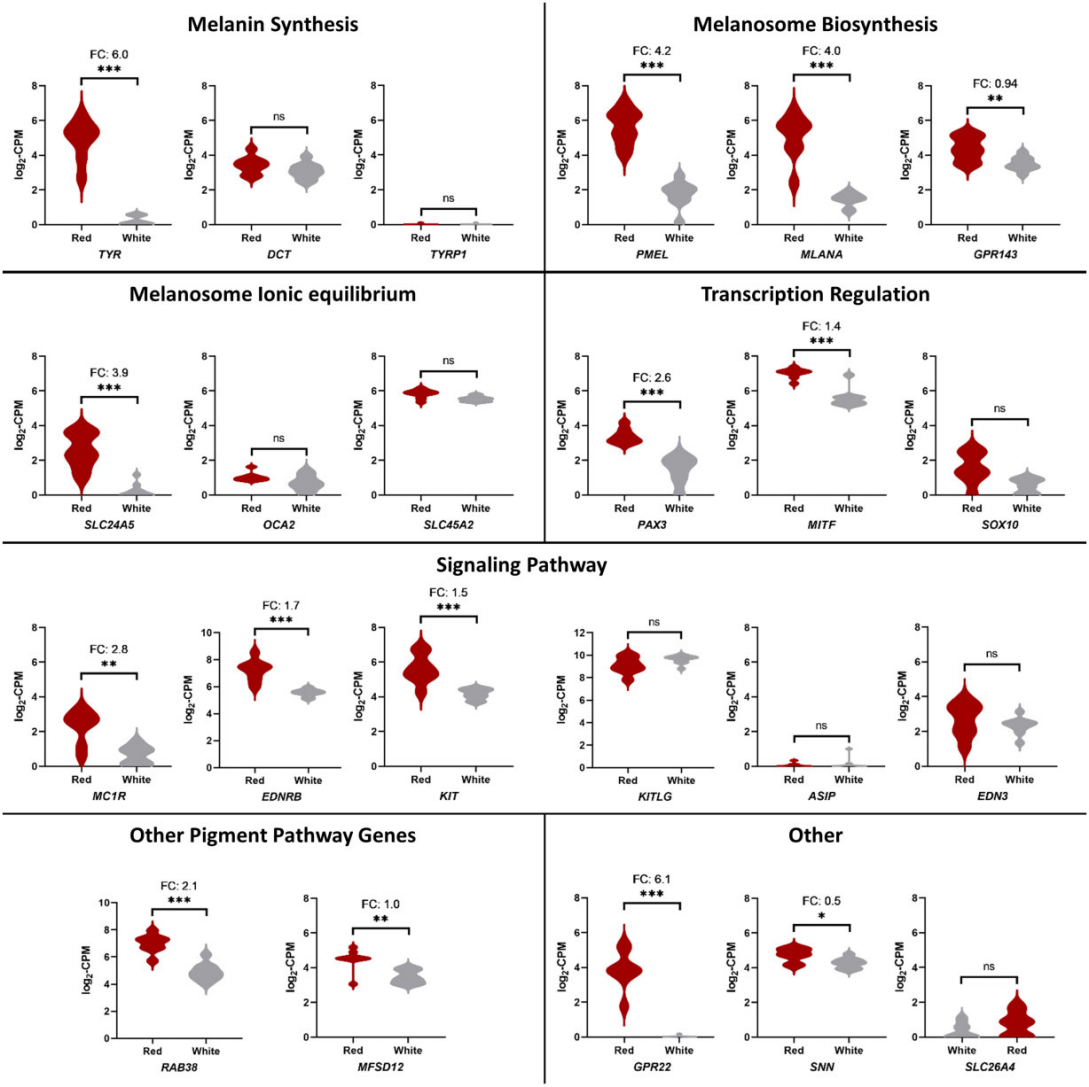
Differential gene expression analysis in Poodle skin tissue. Various genes involved in pigment production were overexpressed in the red Poodles (*N* = 8) compared to the white Poodles (*N* = 9). *GPR22* and *SNN* are also enriched in red Poodle skin.

Gene expression was analyzed in red and white Poodle skin. Poly(A) capture RNAseq was first performed in 3 red Poodles and 1 white Poodle to determine if *SNNL1* was forming novel chimeric transcripts with the nearby genes *GPR22* and *COG5*, and no novel chimeric transcripts were observed in any samples. Overall differences in expression were then analyzed in 8 red and 9 white Poodle skin samples using TagSeq (Supplementary Table 3). Among the most highly overexpressed genes in the red Poodles were several genes involved in melanogenesis, including *TYR*, *PMEL*, *MLANA*, *SLC24A5*, and *MC1R* (Fig. 4). Notably, among genes involved in the production of eumelanin, *TYRP1* was not expressed in either red or white poodle skin, and no changes in expression were observed for *DCT*. The most differentially expressed gene in red Poodle skin was *GPR22*, which was unexpressed in the white Poodles and highly expressed in the red Poodles (FC 6.1; adjusted *P* = 0.00042). *SNN* also had small but significantly increased expression in the red Poodle skin (FC 0.49; adjusted *P* = 0.0315), as did *COG5* (FC 0.41; adjusted *P* = 0.0433). Among the genes with missense variants in the red Poodles, neither *SLC26A4* nor *CDHR3* had sufficient expression in either the red or white Poodle skin to allow for differential expression analysis. However, while low levels of differential expression were observed in *LAMB4* (FC 1.2; adjusted *P* = 0.0024) and *GSAP* (FC-1.1; adjusted *P* = 0.0006). *GPR22*, with a FC of 6.1, was the only gene within the chr18:10–20 mb interval that had greater than 2 FC in expression in the red Poodles.

## Discussion

Here we report the discovery of a recent *SNN* gene retrocopy insertion (*SNNL1*) which is 2.8 kb upstream from *GPR22* and is strongly associated with pheomelanin intensity in Poodles. *SNNL1* was part of a large LD block in Poodles which was identified through quantitative GWAS of pheomelanin colors in Poodles. Missense variants in 5 genes were identified in red Poodles within this region, including a variant in *SLC26A4* which had previously been reported as a candidate for pheomelanin intensity across breeds. However, *SLC26A4* was not expressed in Poodle skin, whereas *GPR22* was identified as the most differentially expressed gene between red and white Poodles. *SNNL1*, which has inserted just upstream of *GPR22*, appears to interfere with regulation of the gene, resulting in ectopic expression of *GPR22* in skin tissue. This study implicates *GPR22* as being involved in the pigment production pathway, and the misregulation of *GPR22* via the insertion of *SNNL1* is likely the causal genetic influence behind red coat color in the Poodle breed.

Retrocopy insertions are a type of large structural variant which can be expressed directly, form chimeric transcripts with nearby genes, or otherwise interrupt the typical expression patterns of nearby genes (Kubiak and Makałowska 2017). The *SNN* retrocopy insertion, *SNNL1*, is a full length copy of the parent gene. Additionally, it has no coding sequence variants differentiating it from the parent gene sequence, indicating that it is likely a recent insertion. *SNN* codes for Stannin, a metal ion binding mitochondrial membrane protein which may be involved in response to toxic substance as well as cell growth and apoptosis (Buck-Koehntop *et al.* 2005; Billingsley *et al.* 2006). No other *SNN* retrocopies are present in an across species database of reference genome retrocopies, indicating that *SNN* is not a commonly retrotransposed gene (Rosikiewicz *et al.* 2017). Interestingly, overall expression of *SNN* was also increased in the red Poodles, which may indicate that *SNNL1* is capable of expression. Notably, multiple recent *FGF4* retrocopy insertions have also been reported in dogs, several of which are expressed and involved in skeletal dysplasias (Parker *et al.* 2009; Brown *et al.* 2017; Batcher *et al.* 2020). In red and apricot Poodles, the insertion of *SNNL1* immediately upstream of *GPR22* is likely affecting regulation of the *GPR22* gene, resulting in its atypical expression in skin tissue. Structural variants, which include retrocopy insertions, have been identified as a major source of gene expression differences which often affect multiple nearby genes (Scott *et al.* 2021). *SNNL1* is inserted within an intron of *COG5*, and while no chimeric reads were observed between the genes, a small increase in expression was observed for *COG5* in the red Poodle skin which may also be a consequence of the retrocopy.

While several genes involved in pheomelanin intensity have been identified across dog breeds, the single-breed GWAS presented in this study only identified the CFA18 locus as significant within the Poodle breed. One of the top SNVs was a missense variant in *SLC26A4* which has previously been associated with pheomelanin intensity across dog breeds, where it was hypothesized to be the causative variant (Slavney *et al.* 2021). The researchers found that the CFA18 locus explained a relatively small % of the total variance in pheomelanin across dog breeds (adjusted *R*^2^ = 0.047), whereas loci on CFA2 and CFA20 explained over 50% of the variance across breeds. However, when looking within a single breed, the Poodle, the CFA18 locus, identified herein as *SNNL1*, explained the majority of the variance between white, cream, apricot, and red Poodles (adjusted *R*^2^ = 0.840). Notably, 70 out of 73 apricot or red poodles had at least 1 copy of *SNNL1*, while none of the 125 white Poodles tested had any copies of *SNNL1*. Only 19% of the cream poodles had 1 copy of *SNNL1*, while the rest had 0 copies. Likely other genes involved in pheomelanin intensity, such as the *MFSD12* dilution variant, explain the differences between white and cream coat colors (Hédan *et al.* 2019). Analysis of the *SLC26A4* missense variant and the *SNNL1* insertion in a larger WGS dataset revealed that they are rare across breeds and were only observed in East Asian dog breeds and village dogs, such as Tibetan Mastiffs, Chow Chows, and, notable for their rich pheomelanin appearance, Qingchuan and Chongqing dogs. It is possible that the CFA18 locus explains a relatively small proportion of the variance in pheomelanin intensity across breeds due to this breed exclusivity. Whereas, within breeds, *SSNL1* may actually mask the effects of other genes involved in pheomelanin intensity. While the CNV upstream of *KITLG* was associated with pheomelanin intensity in Nova Scotia Duck Tolling Retrievers, the *KITLG* CNV was not significantly associated with pheomelanin intensity in Poodles, possibly due to the effects of *SNNL1* within the breed (Bannasch *et al.* 2021).

Pigment production is canonically regulated through the MC1R-tmAC-MITF pathway, which induces changes in expression of pigment genes such as *TYR*, *PMEL*, *MLANA*, *SLC24A5*, *TYRP1*, and *DCT* (Kawakami and Fisher 2017; Bang and Zippin 2021). The MC1R-tmAC-MITF pathway uses the second messenger molecule cyclic adenosine monophosphate (cAMP), and loss of function mutations in the *MC1R* gene lead to impairments in downstream cAMP signaling, resulting in impaired eumelanogenesis and the phenotype known as recessive yellow in dogs (Newton *et al.* 2000). In addition to cAMPs role as a second messenger molecule in the MC1R-tmAC-MITF pathway, tyrosinase itself is also affected by cAMP; reduction in cAMP within the melanosome results in a higher melanosomal pH, leading to greater tyrosinase activity and increased melanogenesis (Zhou *et al.* 2018). The sex hormones estrogen and progesterone have been found to regulate melanin synthesis through the alteration of cAMP signaling, showing that external factors which affect cAMP concentrations can have a downstream effect on melanogenesis (Natale *et al.* 2016). While the pheomelanin-based Poodles used in this study were homozygous for the recessive yellow mutation in *MC1R*, several pigment genes were still observed to be highly overexpressed in red Poodle skin, including *TYR*, *PMEL*, and *MLANA*, indicating upregulation of the melanogenesis pathway in red Poodles. Histopathological analysis in Poodle skin found that melanocytes were present within the hair bulbs of all Poodles, however melanin granules were present in high amounts in the red Poodles, consistent with an upregulation in pigment production in red Poodles. Notably, however, the main drivers behind eumelanogenesis, *TYRP1* and *DCT* (Slominski *et al.* 2004), were not overexpressed in the red Poodles, suggesting specific upregulation in pheomelanogenesis. While *SLC26A4* was not expressed in either red or white Poodle skin, differential expression was observed in several other genes within the red Poodle-associated region on chr18:10–20 mb. However, the degree of differential expression observed in *GPR22* was much higher than any other genes, and was more comparable to the differential expression observed in genes involved in the pigment production pathway. Most notably, a near identical increase in expression was observed for *GPR22* and *TYR* (FC 6.05 and 5.96), which were also the top 2 most differentially expressed genes between red and white Poodle skin.

*GPR22* is an orphan G-coupled protein receptor with a highly restrictive expression pattern in the heart and brain (Adams *et al.* 2008). While *GPR22* knockout mice are viable and grossly indistinguishable from wild-type mice, they may be more susceptible to functional cardiac decompensation following aortic banding (Adams *et al.* 2008). Deregulation of *GPR22* within the zebrafish embryo lead to defects in left-right patterning and resulted in abnormal cilia structure and length, indicating a possible developmental role for *GPR22* (Verleyen *et al.* 2014). *GPR22* has also been implicated in osteoarthritis in humans through GWAS (Kerkhof *et al.* 2010; Evangelou *et al.* 2011). While *GPR22* was absent from healthy cartilage, it was found expressed in damaged cartilage, and overexpression of *GPR22* was also shown to accelerate chondrocyte hypertrophy (Guns *et al.* 2016, 2018). While the *GPR22* ligand is unknown, overexpression of *GPR22* in HEK-293 cells identified that the GPR22 protein signals through the G inhibitory pathway, resulting in inhibition of adenylyl cyclase and a reduction in cAMP (Adams *et al.* 2008). The second messenger cAMP regulates numerous functions in melanocytes, and the abnormal expression of *GPR22* in the melanocytes of red Poodles may have effects on pigmentation through this G inhibitory pathway.

GWAS in dogs often succeed at identifying genomic regions, yet due to the extensive LD in dog breeds, it can be difficult to pinpoint causative mutations. In this study, several nearby missense variants within the pheomelanin associated region on chr18 could not be ruled out by segregation analysis alone. While the expression analysis in the skin was able to rule out the *SLC26A4* and *CDHR3* missense variants, further analysis including quantitative phenotyping in other breeds with the *SNNL1* insertion such as Chow Chows and Tibetan Mastiffs may be required to rule out the other missense variants. Still, the differential expression analysis in Poodle skin highlighted the *SNNL1* insertion and its effects on the expression of *GPR22* as the likely causal mutation behind the red coat color phenotype in Poodles. Further analysis may also quantify the effects of *SNNL1* on eumelanin-based coat patterns, as the upregulation of numerous genes involved in melanogenesis observed in the red Poodles might indicate an effect on eumelanin as well. The identification of another recent, functional retro copy insertion further serves to highlight the often complex nature behind genomic associations, and also shows that novel retrotransposition events continue to contribute to genomic and phenotypic diversity in dogs.

## Supplementary Material

jkac227_Supplementary_Figure_S1

jkac227_Supplementary_Figure_S2

jkac227_Supplementary_Figure_S3

jkac227_Supplementary_Table_S1

jkac227_Supplementary_Table_S2

jkac227_Supplementary_Table_S3

jkac227_Supplementary_Table_S4

## Data Availability

The data underlying this article are available in the Sequence Read Archive at https://www.ncbi.nlm.nih.gov/sra, and can be accessed with BioProject accession PRJNA830895. A description of these files is available in Supplementary Table 4. Supplemental material is available at G3 online.
