# Supplementary figures and images for: An SNN retrocopy insertion upstream of GPR22 is associated with dark red coat color in Poodles
Source: G3 (Bethesda). 2022 Sep 1;12(11):jkac227. doi: 10.1093/g3journal/jkac227 (PMC9635648; doi:10.1093/g3journal/jkac227)

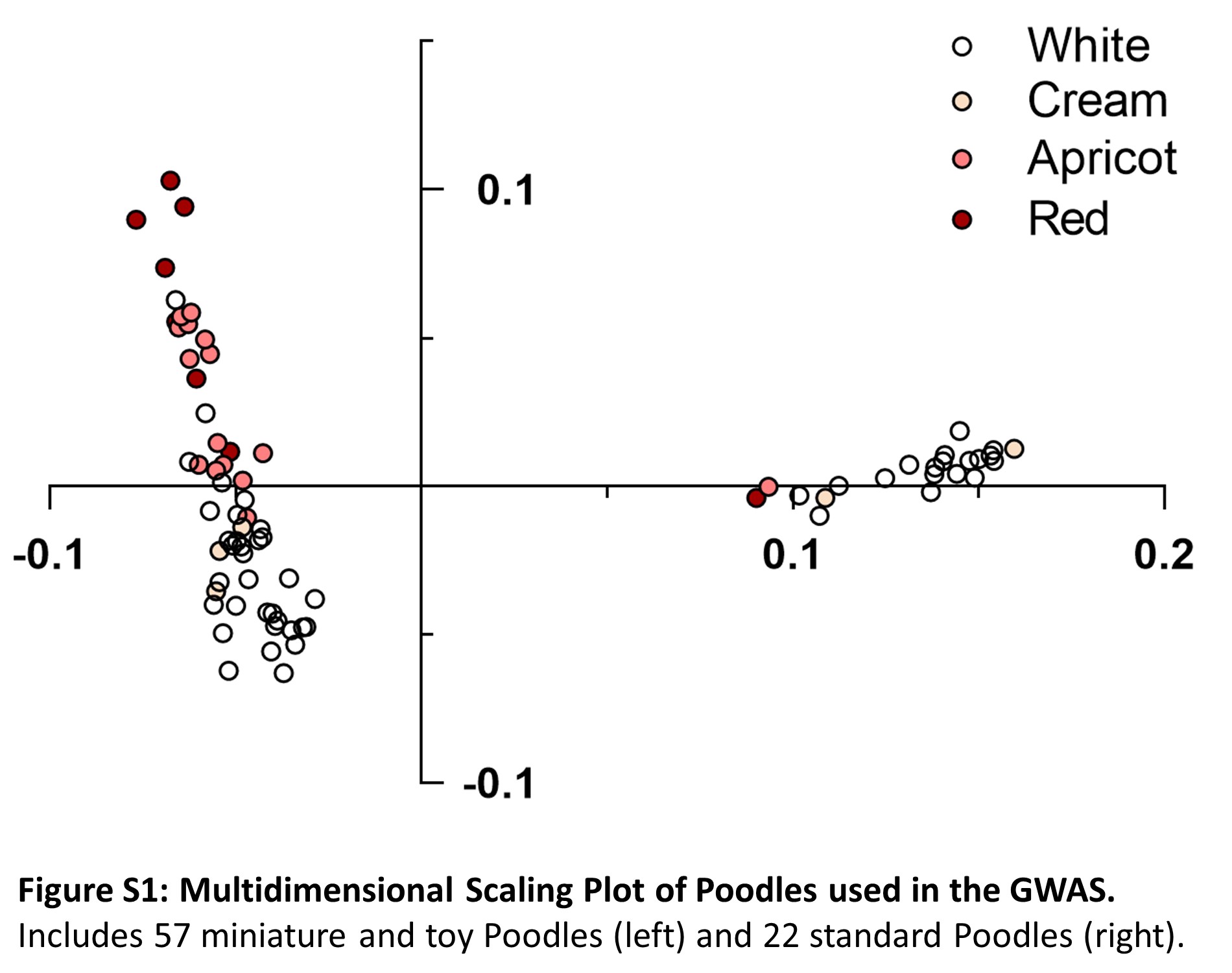

Supplement: jkac227_Supplementary_Figure_S1 [file jkac227_supplementary_figure_s1.jpeg]

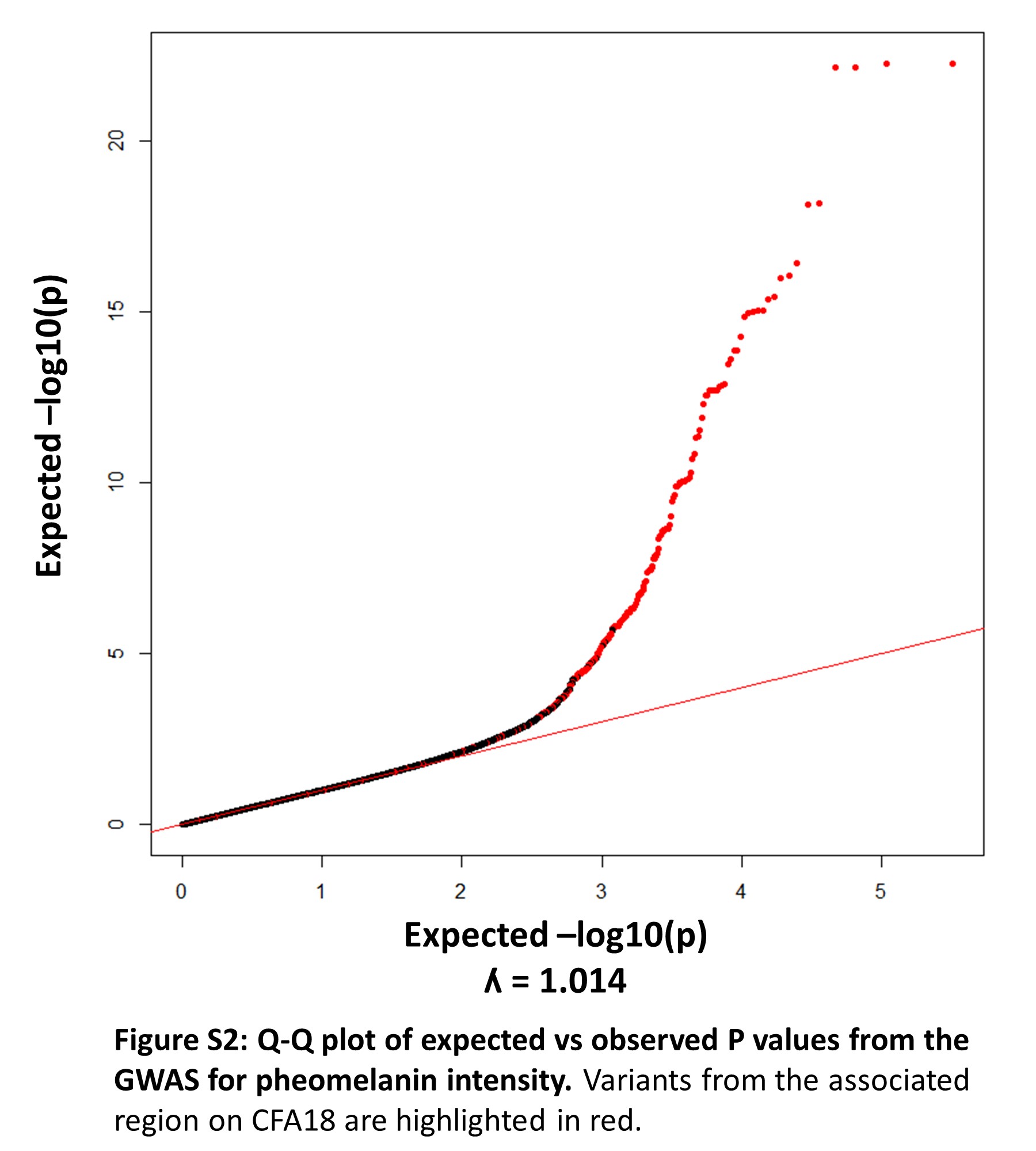

Supplement: jkac227_Supplementary_Figure_S2 [file jkac227_supplementary_figure_s2.jpeg]

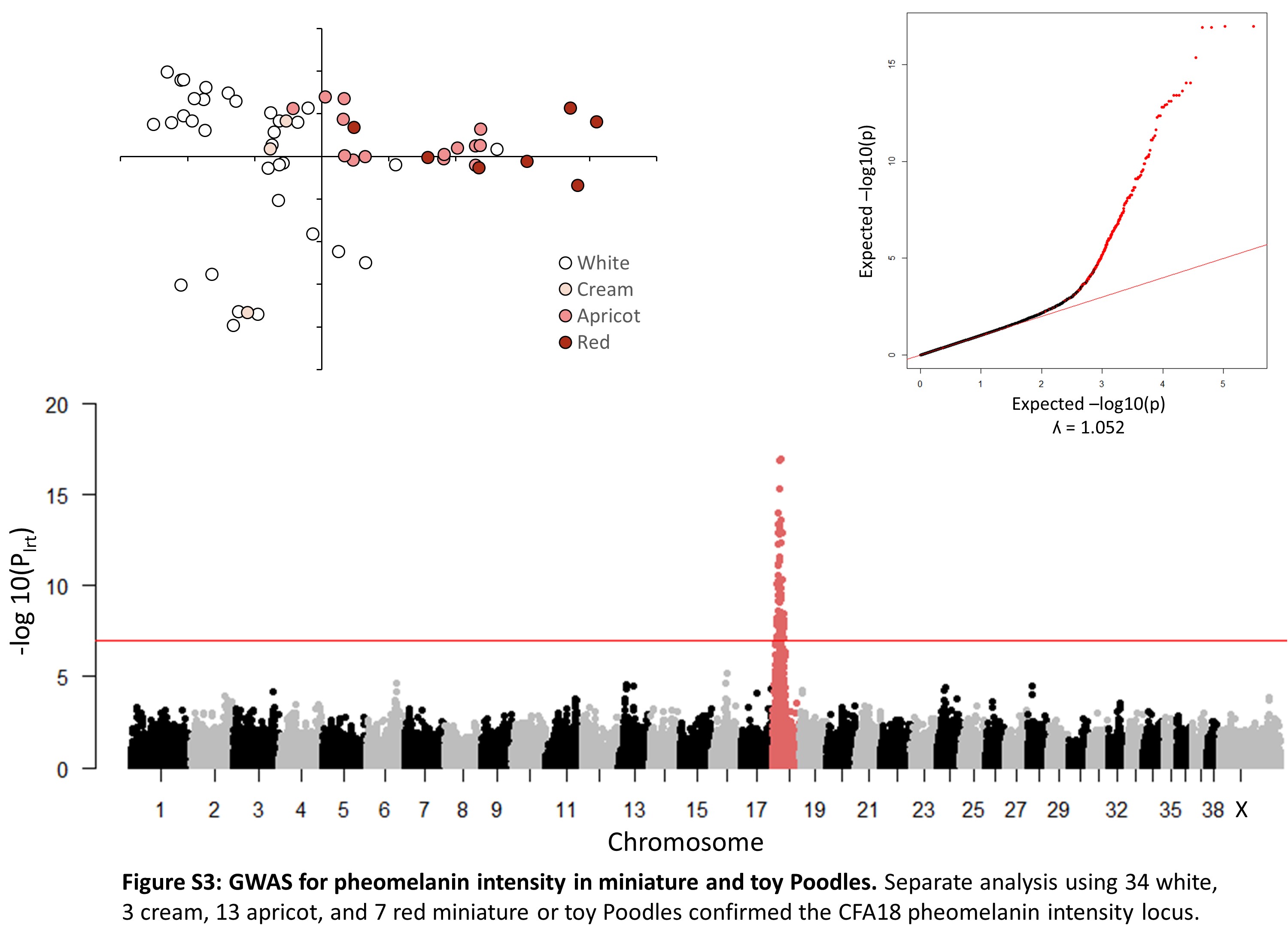

Supplement: jkac227_Supplementary_Figure_S3 [file jkac227_supplementary_figure_s3.jpeg]
